# Supplementary material for: A clinical prediction model to identify children at risk for revisits with serious illness to the emergency department: A prospective multicentre observational study
Source: PLoS One. 2021 Jul 15;16(7):e0254366. doi: 10.1371/journal.pone.0254366 (PMC8281990; doi:10.1371/journal.pone.0254366)
Supplement: S1 Appendix — (PDF) [file pone.0254366.s001.pdf]

S1 Appendix. Sample size estimation

## Methods for sample size estimation

Riley et al. (1,2) proposed to estimate the required sample size for the development and validation of clinical prediction models using

1. Cox Schnell $R^2$ from literature or previous prediction models
   a. Riley et al. also provides a method to calculate the C-S $R^2$ derived from the Nagelkerke's $R^2$ or Area Under the operating characteristic Curve (AUC)
2. The incidence of the outcome
3. Different types of modelling [I.e: in our study: logistic regression models]
4. Number of parameters in model

## Existing prediction models

A systematic search, based on the systematic review by De Vos - Kerkhof et al.(3) yielded [1] relevant paper presenting a prediction model for return visits to the emergency department, reporting appropriate model performances to guide sample size calculation. The selected paper used various modelling techniques including logistic regression. The outcome for the prediction models of this paper is 'Any return visit', and not 'return visit with serious illness'. No prediction model for 'Return visits with serious illness' in children presenting to the emergency department was identified. The variables included in the models by Hu et al. largely agree with our selected variables.

| | Total number of children | Total number of children with index visit | Total number of hospitals | Target population | Definition revisit | Total number of revisits | Total number of revisits with admission | Performance |
|---|---|---|---|---|---|---|---|---|
| Hu et al, 2017 (4) | 457,428 | 125,940 [excluded missing values, pts hospitalised within 24 hrs after ED visit; one visit per patient] | National health insurance database, Taiwan, between 1998 and 2009 | Children =/<18 years, consecutive | <72 hrs, any RV | 6,282 (5.0%) | 1,834 (1.5%) | AUC 0.718 (LR method) |

ED emergency department; RV revisit

## Sample sizes

As calculated by the [pmsampsize] function in R:
https://cran.r-project.org/web/packages/pmsampsize/index.html

| | AUC | C-S $R^2$ | incidence | parameters | Total sample size | Number of events | Events per predictor parameter (EPP) |
|---|---|---|---|---|---|---|---|
| Scenario 1: Hu et al. (1) | 0.718 | 0.06 | 1,026/98561 | 33 | 5,799 | 61 | 1.83 |
| Scenario 2: lower C-S $R^2$ | - | 0.02 | 1,026/98561 | 33 | 14,685 | 153 | 4.63 |

C-S Cox Schnell $R^2$

**References:**


1. Riley RD, Snell KI, Ensor J, Burke DL, Harrell FEJ, Moons KG, et al. Minimum sample size for developing a multivariable prediction model: PART II - binary and time-to-event outcomes. *Stat Med* 2019 Mar;**38**(7):1276–96.
2. Riley RD, Ensor J, Snell KIE, Harrell FE, Martin GP, Reitsma JB, et al. Calculating the sample size required for developing a clinical prediction model. *BMJ* [Internet] 2020;**368**. Available from: https://www.bmj.com/content/368/bmj.m441
3. De Vos-Kerkhof E, Geurts DHF, Wiggers M, Moll HA, Oostenbrink R. Tools for “safety netting” in common paediatric illnesses: A systematic review in emergency care. *Arch Dis Child* 2016 Feb;**101**(2):131–9.
4. Hu YH, Tai CT, Chen SCC, Lee HW, Sung SF. Predicting return visits to the emergency department for pediatric patients: Applying supervised learning techniques to the Taiwan National Health Insurance Research Database. *Comput Methods Programs Biomed* 2017 Jun;**144**:105–12.
