# Supplementary material for: A clinical prediction model to identify children at risk for revisits with serious illness to the emergency department: A prospective multicentre observational study
Source: PLoS One. 2021 Jul 15;16(7):e0254366. doi: 10.1371/journal.pone.0254366 (PMC8281990; doi:10.1371/journal.pone.0254366)
Supplement: S1 Table — (PDF) [file pone.0254366.s002.pdf]

S1 Table. Definitions of presenting symptoms and lifesaving interventions

Main categories for presenting problem	*Manchester Triage System (MTS) flowcharts*
Shortness of breath	Asthma<br>Shortness of breath in adults<br>Shortness of breath in children
ENT problems	Ear problems<br>Sore throat
Gastro-intestinal problems	Abdominal pain in adults<br>Abdominal pain in children<br>Diarrhoea and vomiting<br>GI bleeding
Neurological problem	Behaving strangely<br>Fits<br>Headache<br>Irritable child<br>Neck pain
Unwell child	Crying baby<br>Unwell child<br>Worried parent
Urological problems	Testicular pain<br>Urinary problems
Rash	Rashes
Abscess and soft tissue infection	Abscesses and local infections
Trauma	Assault<br>Falls<br>Head injury<br>Limb problems<br>Major trauma<br>Torso injury
Wounds	Bites and stings<br>Burns and scalds<br>Wounds
Other	Allergy<br>Apparently drunk<br>Back pain<br>Chest pain<br>Collapsed adult<br>Dental problems<br>Diabetes<br>Exposure to chemicals<br>Eye problems<br>Facial problems<br>Foreign body<br>Limping child<br>Mental illness<br>Overdose and poisoning<br>Palpitations<br>Pregnancy<br>PV bleeding<br>Self-harm<br>Sexually acquired infection<br>Unwell adult<br>Major incidents – primary<br>Major incidents – secondary<br>General / Other

Immediate lifesaving interventions performed in the emergency department (ED) included:

1. airway and breathing support (i.e., non-rebreathing mask, high flow oxygen, intubation and mechanical ventilation),
2. haemodynamic support (i.e., significant fluid bolus >20 mls/kg, blood transfusion),
3. other emergency procedures (i.e., defibrillation, chest needle decompression, pericardiocentesis, or open thoracotomy),
4. emergency medications (i.e., atropine, adenosine, inotropes, epinephrine, naloxone, dextrose in case of hypoglycaemia).
