# Supplementary material for: A clinical prediction model to identify children at risk for revisits with serious illness to the emergency department: A prospective multicentre observational study
Source: PLoS One. 2021 Jul 15;16(7):e0254366. doi: 10.1371/journal.pone.0254366 (PMC8281990; doi:10.1371/journal.pone.0254366)
Supplement: S2 Table — (PDF) [file pone.0254366.s003.pdf]

S2 Table. Characteristics of the index visit of children admitted to PICU at the time of revisit

23 children were admitted to PICU when they returned to the ED; one child died. These cases had varying levels of urgency and severity at the time of their index visit, with a mean risk prediction of 3.7% using the clinical model (range 0.1% - 7.3%; 4 children had a predicted risk below prevalence of <2.0%). These children had a mean risk of 3.7% for the extended model (range 0.1% - 13.1%; 5 children risk <2.0%). 9/23 (39%) cases were aged <1 years old. One child died at return to the ED with an original risk estimate of 2.1%; extended model 1.8%.

		N (%)
Age	Years, median (range)	1.27 (0.04 – 8.36)
Gender		
MTS Flowchart for presenting problem	Shortness of breath	9 (39%)
	Vomiting and diarrhea	3 (13%)
	Abdominal pain	1 (4%)
	Unwell child	4 (17%)
	Fits	1 (4%)
	Worried parent	3 (13%)
	Chest pain	1 (4%)
	Rashes	2 (8%)
MTS triage Urgency classification	Emergent	0
	Very urgent	7 (29%)
	Urgent	9 (38%)
	standard	8 (33%)
	Non-urgent	0
Vital signs	Tachycardia	8 (33%)
	Tachypnoea	7 (29%)
	Temperature >37.9 degrees Celsius	12 (50%)
	Oxygen saturations <94%	1 (4%)
	Capillary refill ≥ 3 s	0
	Level of consciousness: decreased	4 (17%)
