# Supplementary material for: A clinical prediction model to identify children at risk for revisits with serious illness to the emergency department: A prospective multicentre observational study
Source: PLoS One. 2021 Jul 15;16(7):e0254366. doi: 10.1371/journal.pone.0254366 (PMC8281990; doi:10.1371/journal.pone.0254366)
Supplement: S3 Table — (PDF) [file pone.0254366.s004.pdf]

S3 Table. Clinical prediction models for the three hospitals with data on co-morbidity available

| | | **Odds Ratio+ (95% CI)** |
|---|---|---|
| **Co-morbidity** | **Non-complex** | 2.36 (1.82 – 3.05) |
| | **Complex** | 3.39 (2.67 – 4.29) |
| | **None** | *reference* |
| hospital | Erasmus MC | 2.24 (1.74 – 2.88) |
| | St Mary's Hospital | 1.76 (1.32 – 2.35) |
| | Medizinische Universitaet Wien | *reference* |
| Day and time of arrival | Weekday evenings | 1.23 (0.97 – 1.56) |
| | Weekday nights | 1.08 (0.76 – 1.53) |
| | Weekend days | 1.24 (0.97 – 1.59) |
| | Weekend evenings | 0.91 (0.63 – 1.31) |
| | Weekend nights | 1.32 (0.84– 2.07) |
| | Weekdays | *reference* |
| Season | Winter | 1.14 (0.91 – 1.44) |
| | Spring | 0.98 (0.75 – 1.28) |
| | Summer | 0.96 (0.75 – 1.24) |
| | Autumn | *reference* |
| Age | <1 years | 1.98 (1.44 – 2.72) |
| | 1 - <2 years | 1.41 (1.00– 1.97) |
| | 2 - <5 years | 1.00 (0.73 – 1.38) |
| | 5 - <12 years | 0.83 (0.61 – 1.13) |
| | 12 – 16 years | *reference* |
| Sex | Female | 0.83 (0.69 – 0.99) |
| Presenting problem | Shortness of breath | 1.84 (1.16 – 2.94) |
| | ENT problems | 1.00 (0.52 – 1.93) |
| | Gastro-intestinal problems | 1.93 (1.26 – 2.96) |
| | Neurological problem | 2.22 (1.35 – 3.66) |
| | Unwell child | 1.42 (0.92 – 2.17) |
| | Urological problems | 0.84 (0.41 – 1.72) |
| | Rash | 1.06 (0.57 – 2.00) |
| | Abscess and soft tissue infection | 1.98 (0.97 – 4.06) |
| | Wounds | 0.31 (0.17 – 0.56) |
| | Trauma | 0.79 (0.38 – 1.64) |
| | Other | *Reference* |
| Triage urgency | Emergent / very urgent | 1.14 (0.79 – 1.65) |
| | urgent | 1.53 (1.24 – 1.88) |
| | Standard / non-urgent | *reference* |
| Tachycardia | present | 1.24 (0.97 – 1.60) |
| Tachypnoea | present | 1.17 (0.91 – 1.51) |
| Temperature | >= 38.0 degrees Celsius | 0.96 (0.74 – 1.24) |
| Oxygen saturations | Oxygen saturation <94% | 1.25 (0.66 – 2.37) |
| Level of consciousness | Reduced | 1.38 (0.77 – 2.48) |
| Laboratory tests | Any | 1.75 (1.42 – 2.16) |
| Imaging | Any | 1.34 (1.03 – 1.74) |
| IV medication or fluids | Any | 1.04 (0.67 – 1.61) |

+: aOR: adjusted odds ratio, with: Serious illness ~ hospital + time and day of arrival + season + age + gender + presenting problem + triage urgency classification + presence of tachycardia + presence of tachypnoea + temperature ≧ 38.0 ºC + Oxygen saturations < 94% + level of consciousness (reduced) + any laboratory tests performed + any imaging performed + any iv fluids and/or medications given

Co-morbidity data available for Medizinische Universitaet Wien, Vienna, Austria; Erasmus MC, Rotterdam, the Netherlands; St Mary's Hospital, London, United Kingdom

CI confidence interval; IV intravenous; OR Odds Ratio
