# Supplementary material for: A clinical prediction model to identify children at risk for revisits with serious illness to the emergency department: A prospective multicentre observational study
Source: PLoS One. 2021 Jul 15;16(7):e0254366. doi: 10.1371/journal.pone.0254366 (PMC8281990; doi:10.1371/journal.pone.0254366)
Supplement: S4 Table — (PDF) [file pone.0254366.s005.pdf]

S4 Table. Regression coefficients of the final clinical prediction models

		Coefficient	Standard error	Coefficient	Standard error
		Clinical model		***Extended model***	
Day and time of arrival	Weekday evenings	.006	.087	.039	.088
	Weekday nights	.201	.113	.233	.114
	Weekend days	.180	.093	.193	.094
	Weekend evenings	.057	.121	.092	.122
	Weekend nights	.251	.153	.275	.153
	Weekdays	*reference*		*reference*	
Season	Winter	.159	.086	.158	.086
	Spring	.139	.091	.133	.091
	Summer	-.012	.095	-.040	.095
	Autumn	*reference*		*reference*	
Age	<1 years	.792	.121	.979	.123
	1 - <2 years	.307	.131	.490	.133
	2 - <5 years	.146	.121	.286	.122
	5 - <12 years	.006	.118	.075	.118
	12 – 16 years	*reference*		*reference*	
Sex	Female	-.067	.065	-.071	.065
Presenting problem	Shortness of breath	.634	.177	.646	.177
	ENT problems	-.185	.229	-.162	.230
	Gastro-intestinal problems	.960	.159	.838	.161
	Neurological problem	.840	.200	.789	.200
	Unwell child	.571	.161	.493	.162
	Urological problems	.231	.273	.070	.275
	Rash	-.197	.228	-.115	.229
	Abscess and soft tissue infection	.792	.265	.795	.265
	Wounds	-.737	.188	-.848	.193
	Trauma	-1.035	.336	-.896	.336
	Other	*reference*		*reference*	
Triage urgency	Emergent / very urgent	.654	.120	.518	.120
	urgent	.603	.077	.511	.078
	Standard / non-urgent	*reference*		*reference*	
Tachycardia	present	.266	.088	.251	.089
Tachypnoea	present	.169	.088	.165	.088
Temperature	>= 38.0 degrees Celsius	-.030	.098	-.089	.099
Oxygen saturations	Oxygen saturation <94%	.406	.233	.355	.233
Level of consciousness	Reduced	.432	.256	.335	.257
Laboratory tests	Any	-	-	.454	.079
Imaging	Any	-	-	.478	.088
IV medication or fluids	Any	-	-	.637	.110
Intercept		-5.541		-5.869	

Legend:

Clinical model:

Linear predictor = -5.541 + 0.006 * Time of presentation: Weekday evenings + 0.201 * Time of presentation: Weekday nights + 0.180 * Time of presentation: Weekend days + 0.057 * Time of presentation: Weekend

evenings + 0.251 * Time of presentation: Weekend nights (*reference*:Time of presentation: Weekdays) + 0.159 * Season: Winter + 0.139 * Season: Spring + -0.012 * Season: Summer (*reference*: Season: Autumn) + 0.792 * Age: <1 years + 0.307 * Age: 1 - <2 years + 0.146 * Age: 2 - <5 years + 0.006 * Age: 5 - <12 years (*reference*: Age: 12 – 16 years) + -0.067 * Sex: Female + 0.634 * Presenting problem: Shortness of breath + -0.185 * Presenting problem: ENT problems + 0.960 * Presenting problem: Gastro-intestinal problems + 0.840 * Presenting problem: Neurological problem + 0.571 * Presenting problem: Unwell child + 0.231 * Presenting problem: Urological problems + -0.197 * Presenting problem: Rash + 0.792 * Presenting problem: Abscess and soft tissue infection + -0.737 * Presenting problem: Wounds + -1.035 * Presenting problem: Trauma (*reference*: Presenting problem: Other) + 0.654 * Triage urgency: Emergent / very urgent + 0.603 * Triage urgency: urgent (*reference*: Triage urgency: Standard / non-urgent) + 0.266 * Tachycardia present + 0.169 * Tachypnoea present + -0.030 * Temperature >= 38.0 degrees Celsius + 0.406 * Oxygen saturation <94% + .432 * Level of consciousness - Reduced

Extended model:
Linear predictor: -5.869 + 0.039 * Time of arrival: Weekday evenings + 0.233 * Time of arrival: Weekday nights + 0.193 * Time of arrival: Weekend days + 0.092 * Time of arrival: Weekend evenings + 0.275 * Time of arrival: Weekend nights (*reference*: Time of arrival: Weekdays) + 0.158 * Season: Winter + 0.133 * Season: Spring + -0.040 * Season: Summer (*reference*: Season: Autumn) + 0.979 * Age: <1 years + 0.490 * Age: 1 - <2 years + 0.286 * Age: 2 - <5 years + 0.075 * Age: 5 - <12 years (*reference*: Age: 12 – 16 years) + -0.071 * Sex Female + 0.646 * Presenting problem: Shortness of breath + -0.162 * Presenting problem: ENT problems + 0.838 * Presenting problem: Gastro-intestinal problems + 0.789 * Presenting problem: Neurological problem + 0.493 * Presenting problem: Unwell child + 0.070 * Presenting problem: Urological problems + -0.115 * Presenting problem: Rash + 0.795 * Presenting problem: Abscess and soft tissue infection + -0.848 * Presenting problem: Wounds + -0.896 * Presenting problem: Trauma (*reference*: Presenting problem: Other) + 0.518 * Triage urgency Emergent / very urgent + 0.511 * Triage urgency urgent (*reference*: Triage urgency Standard / non-urgent) + 0.251 * Tachycardia present + 0.165 * Tachypnoea present + -0.089 * Temperature >= 38.0 degrees Celsius + 0.355 * Oxygen saturation <94% + 0.335 * Level of consciousness - Reduced + 0.454 * Laboratory tests + 0.478 * Imaging + 0.637 * IV medication or fluids

To calculate risk prediction: 1 / 1 + (exp (-linear predictor))

CI confidence interval; ENT Ear nose and throat; IV intravenous;
