# Supplementary material for: A clinical prediction model to identify children at risk for revisits with serious illness to the emergency department: A prospective multicentre observational study
Source: PLoS One. 2021 Jul 15;16(7):e0254366. doi: 10.1371/journal.pone.0254366 (PMC8281990; doi:10.1371/journal.pone.0254366)
Supplement: S5 Table — (PDF) [file pone.0254366.s006.pdf]

S5 Table. Percentiles of risk

	Probability of return visits with serious illness:	
Percentiles	Clinical model	Extended model
≦1st	0.0021241	0.0018643
<1st - ≦5th	0.0030213	0.0026209
>5th - ≦10th	0.0036470	0.0033851
>10th - ≦20th	0.0049767	0.0044562
>20th - ≦30th	0.0061339	0.0055798
>30th - ≦40th	0.0080794	0.0078344
>40th - ≦50th	0.0119793	0.0107996
>50th - ≦60th	0.0157816	0.0142450
>60th - ≦70th	0.0206832	0.0186698
>70th - ≦80th	0.0267819	0.0244827
>80th - ≦90th	0.0365092	0.0346855
>90th - ≦95th	0.0447449	0.0465193
>95th - ≦99th	0.0637588	0.0829442
>99th	> 0.0637588	> 0.0829442

Upper risk threshold for probabilities displayed (to convert to %: *100%)
