# Supplementary material for: A clinical prediction model to identify children at risk for revisits with serious illness to the emergency department: A prospective multicentre observational study
Source: PLoS One. 2021 Jul 15;16(7):e0254366. doi: 10.1371/journal.pone.0254366 (PMC8281990; doi:10.1371/journal.pone.0254366)
Supplement: S6 Table — (PDF) [file pone.0254366.s007.pdf]

S6 Table. Variance inflation factors

		Variance inflation factor
Hospital	Erasmus MC	2.613684
	Maasstad Hospital	2.192976
	St Mary's Hospital	1.935705
	Hospital Fernando da Fonseca	2.805936
	Medizinische Universitaet Wien	*reference*
Day and time of arrival	Weekday evenings	1.218736
	Weekday nights	1.154591
	Weekend days	1.186321
	Weekend evenings	1.118119
	Weekend nights	1.082367
	Weekdays	*reference*
Season	Winter	1.481123
	Spring	1.512887
	Summer	1.416671
	Autumn	*reference*
Age	<1 year	3.061199
	1 - <2 years	2.226779
	2 - <5 years	2.558803
	5 - <12 years	2.424041
	12 – 16 years	*reference*
Gender	Female	1.015633
Presenting problem	Shortness of breath	4.594029
	ENT problems	1.678287
	Gastro-intestinal problems	4.652088
	Neurological problem	2.074286
	Unwell child	5.232040
	Urological problems	1.387111
	Rash	1.666545
	Abscess and soft tissue infection	1.409264
	Wounds	2.481816
	Trauma	1.225583
	Other	*reference*
Triage urgency	Emergent / very urgent	1.851208
	urgent	1.448917
	Standard / non-urgent	*reference*
Tachycardia	present	1.258764
Tachypnoea	present	1.187665
Temperature	>= 38.0 degrees Celsius	1.346491
Oxygen saturations	Oxygen saturation <94%	1.060575
Level of consciousness	Reduced	1.074351
Laboratory tests	Any	1.252935
Imaging	Any	1.110406
IV medication or fluids	Any	1.382696
