# Supplementary material for: A clinical prediction model to identify children at risk for revisits with serious illness to the emergency department: A prospective multicentre observational study
Source: PLoS One. 2021 Jul 15;16(7):e0254366. doi: 10.1371/journal.pone.0254366 (PMC8281990; doi:10.1371/journal.pone.0254366)
Supplement: S1 Fig — (PDF) [file pone.0254366.s008.pdf]

S1 Fig. Area Under the Receiver Operating Curve (AUC) for validation cohorts

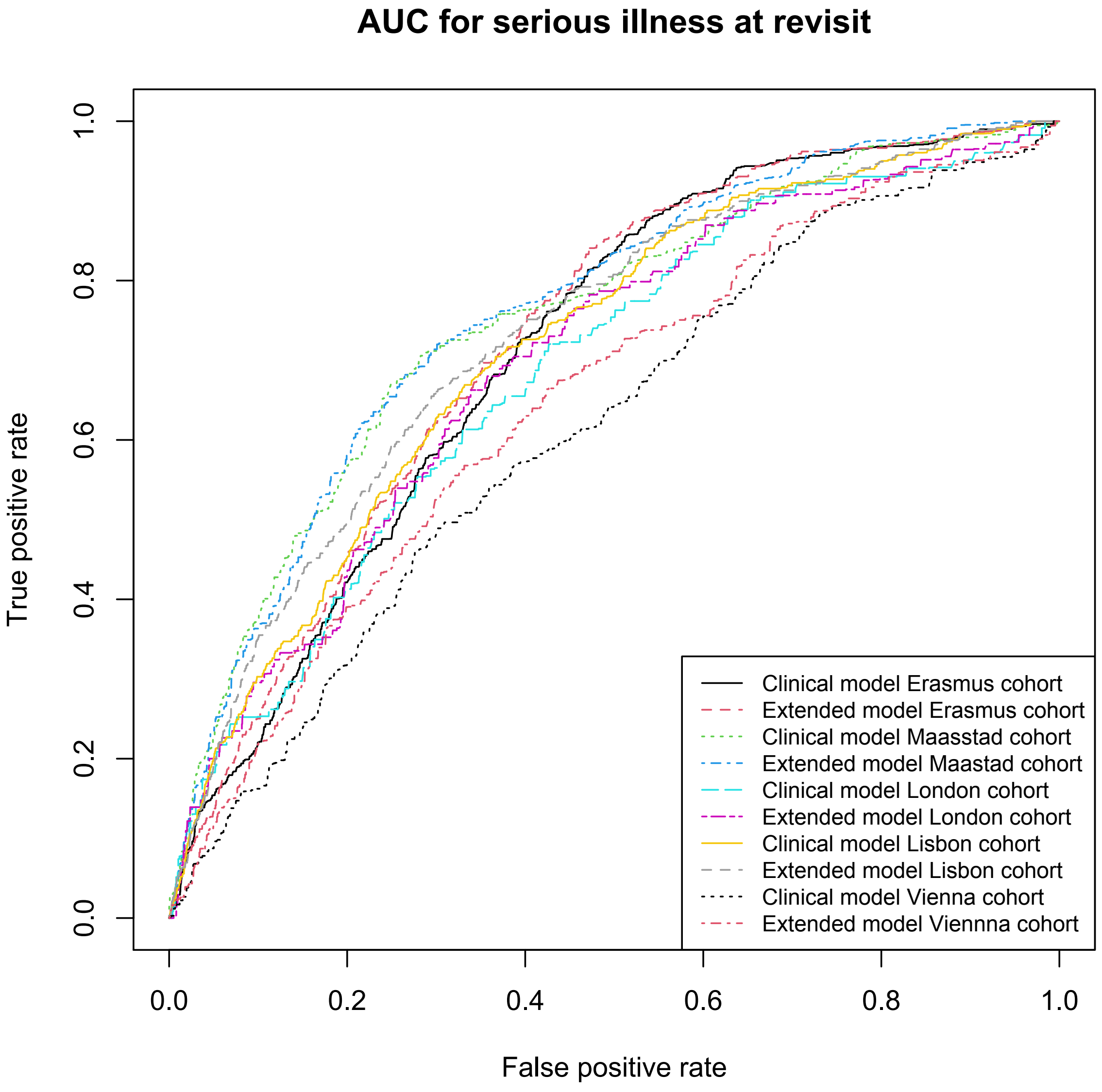

Legend:
AUC curves for both the clinical and extended models from the cross-validation ("leave-one-out" approach) studies of all five cohorts.
