# Supplementary material for: A clinical prediction model to identify children at risk for revisits with serious illness to the emergency department: A prospective multicentre observational study
Source: PLoS One. 2021 Jul 15;16(7):e0254366. doi: 10.1371/journal.pone.0254366 (PMC8281990; doi:10.1371/journal.pone.0254366)
Supplement: S2 Fig — (PDF) [file pone.0254366.s009.pdf]

S2 Fig. Forest plots for cross-validationArea Under the Receiver Operating Curves (AUC)

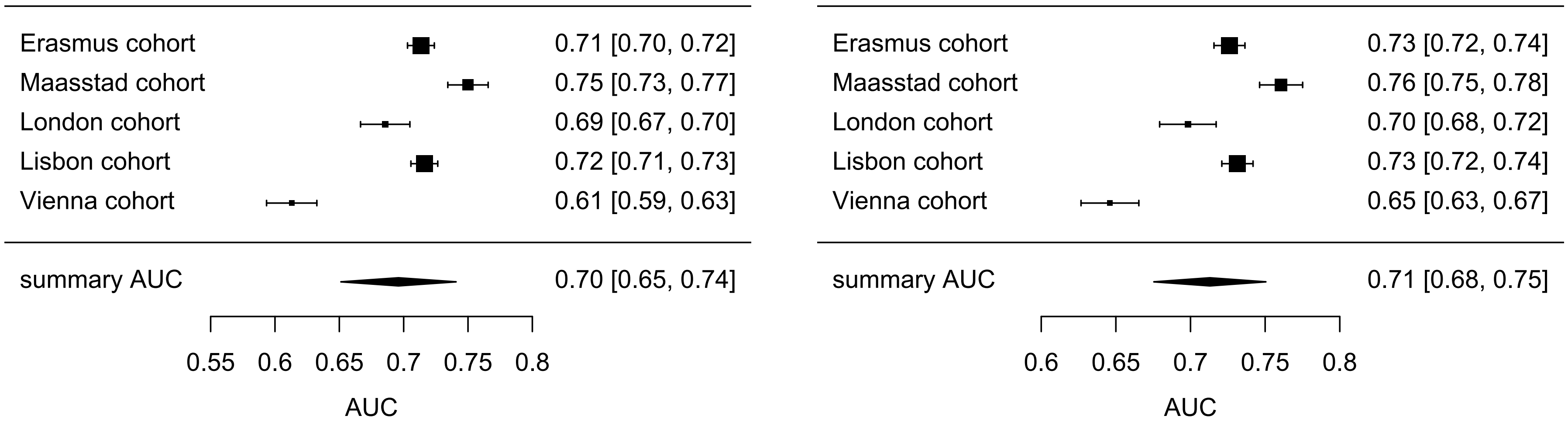

Legend:

Forest plot of random effects model of the cross-validation AUCs of the clinical models (left) and extended models (right) for our five cohorts (y-axis). The black squares show the mean AUC values with the 95% confidence intervals on the x-axis. Overall summary AUC and confidence interval are shows as black diamond. For clinical model $I^2 = 98.06\%$, test for heterogeneity $Q(df = 4) = 126.8405$, p-value < 0.0001; for extended model $I^2 = 97.34\%$, test for heterogeneity $Q(df = 4) = 96.3497$, p-value < 0.0001
