# Supplementary material for: A clinical prediction model to identify children at risk for revisits with serious illness to the emergency department: A prospective multicentre observational study
Source: PLoS One. 2021 Jul 15;16(7):e0254366. doi: 10.1371/journal.pone.0254366 (PMC8281990; doi:10.1371/journal.pone.0254366)
Supplement: S3 Fig — (PDF) [file pone.0254366.s010.pdf]

S3 Fig. Forest plots for cross validation calibration slopes

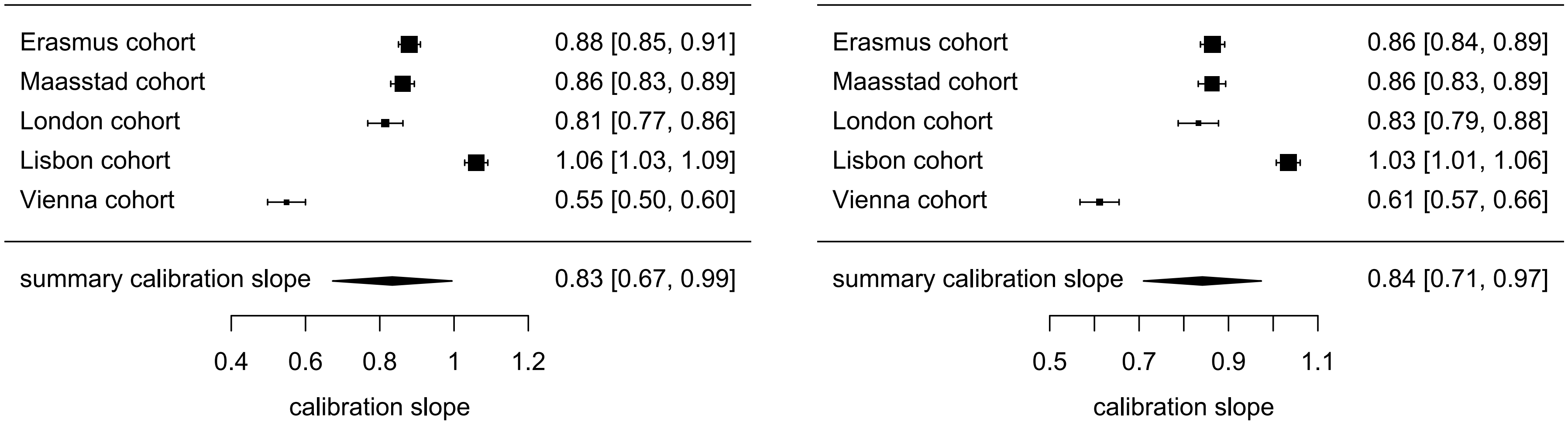

Legend:

Forest plot of random effects mixed model of the cross-validation calibration slopes of the clinical models (left) and extended models (right) for our five cohorts (y-axis). The black squares show the mean calibration slope with the 95% confidence intervals on the x-axis. Overall summary calibration slope and confidence interval are shows as black diamond. For clinical model I^2 = 98.99%, test for heterogeneity Q(df = 4) =298.5620, p-value < 0.0001; for extended model I^2 = 98.76%, test for heterogeneity Q(df = 4) = 278.1791, p-value < 0.0001
