# Supplementary material for: A clinical prediction model to identify children at risk for revisits with serious illness to the emergency department: A prospective multicentre observational study
Source: PLoS One. 2021 Jul 15;16(7):e0254366. doi: 10.1371/journal.pone.0254366 (PMC8281990; doi:10.1371/journal.pone.0254366)

S4 Fig. Calibration plots for cross-validation cohorts

Legend:

Calibration plots show the predicted risk (x-axis) and the observed frequencies (y-axis) of return visits with serious illness for both the clinical (left) and extended model (right) for each of the cross-validation studies of the five cohorts. This means that the prediction model derived in four cohorts is validated in the remaining fifth cohort, of which we present the calibration plots here. The triangles represent the mean (predicted vs. observed) risk estimates by quintiles of predicted risks. The dashed diagonal line represents ideal calibration. The distribution of the predicted risks of patients with serious illness and patients without serious illness is shown in the bottom of the graph, parallel to the x- axis; frequencies are included in table 2.

Cross-validation for hospital 1: Erasmus cohort
Left: clinical model
Right: extended model

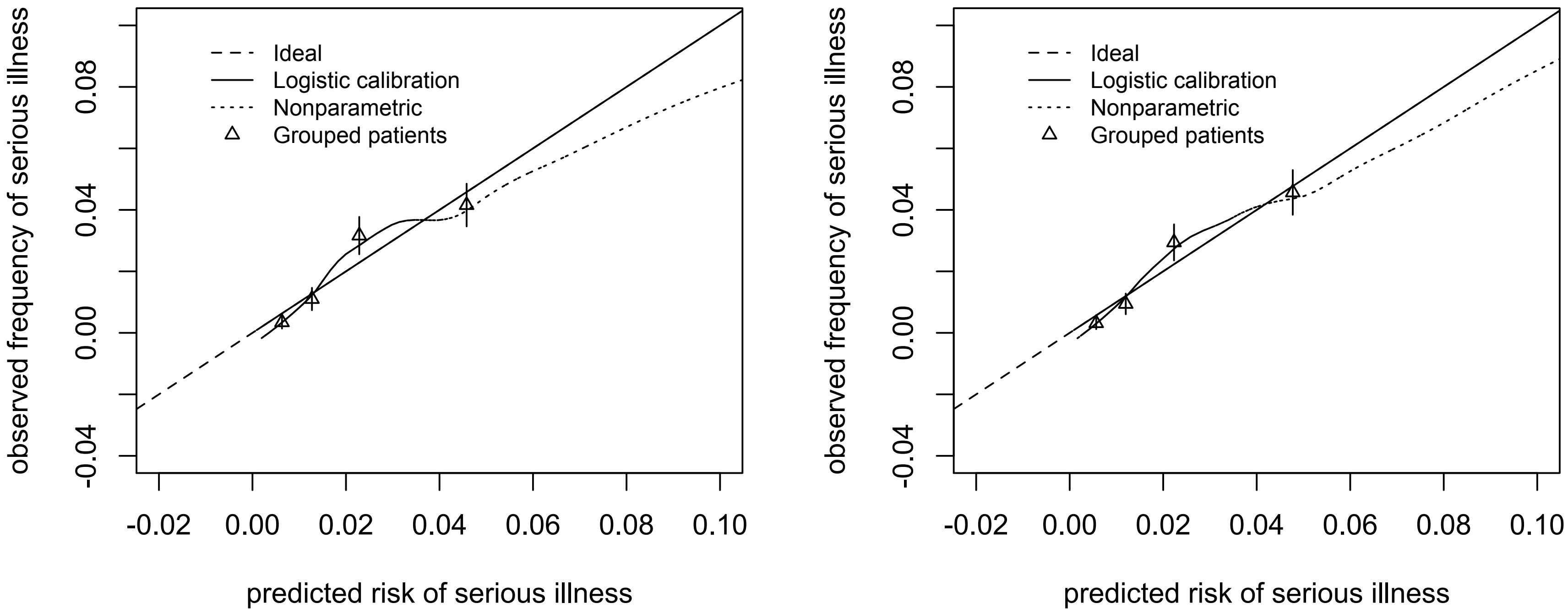

Cross-validation for hospital 2: Maastad cohort

Left: clinical model

Right: extended model

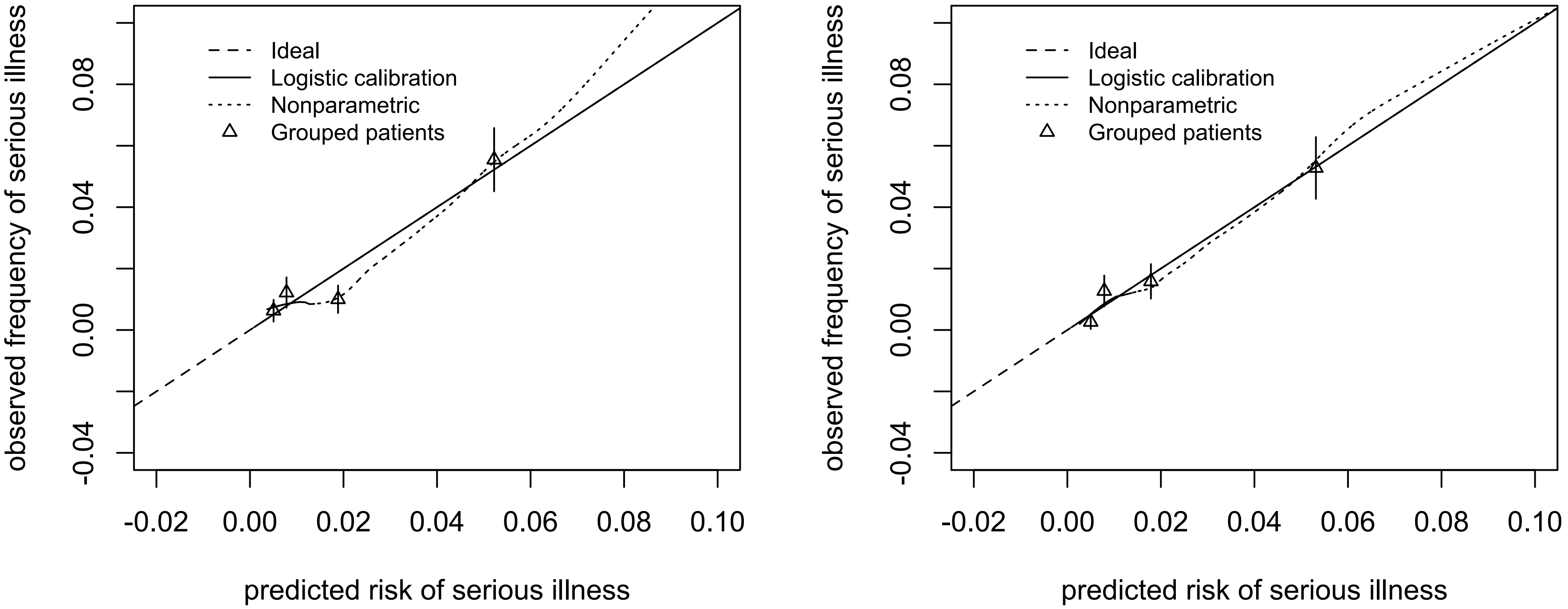

Cross-validation for hospital 3: London cohort
Left: clinical model
Right: extended model

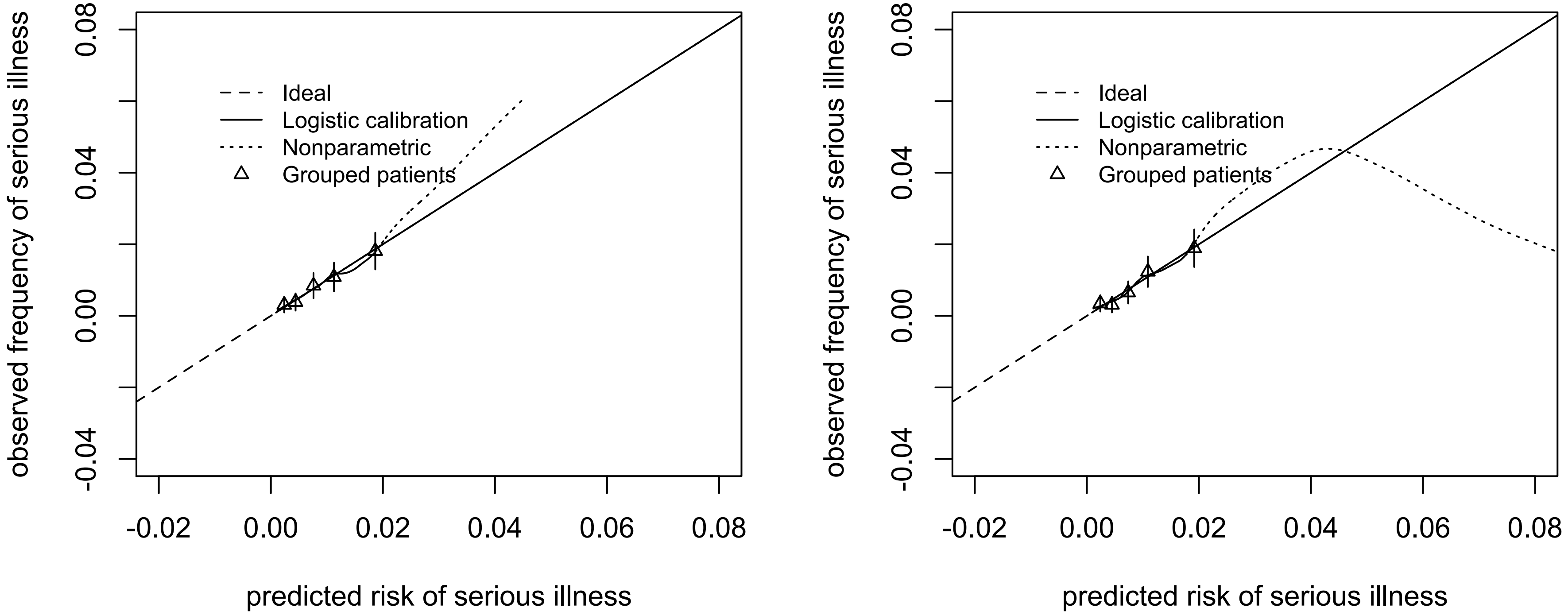

Cross-validation for hospital 4: Lisbon cohort
Left: clinical model
Right: extended model

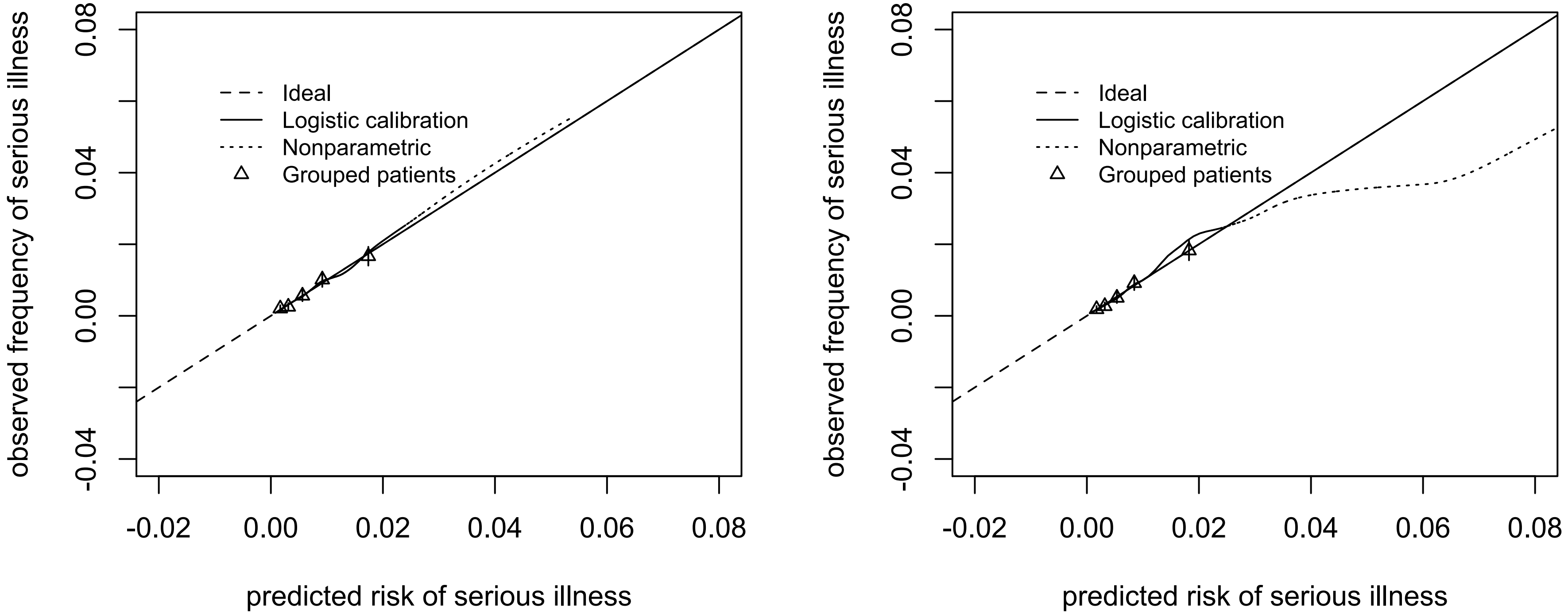

Cross-validation for hospital 5: Vienna cohort
Left: clinical model
Right: extended model

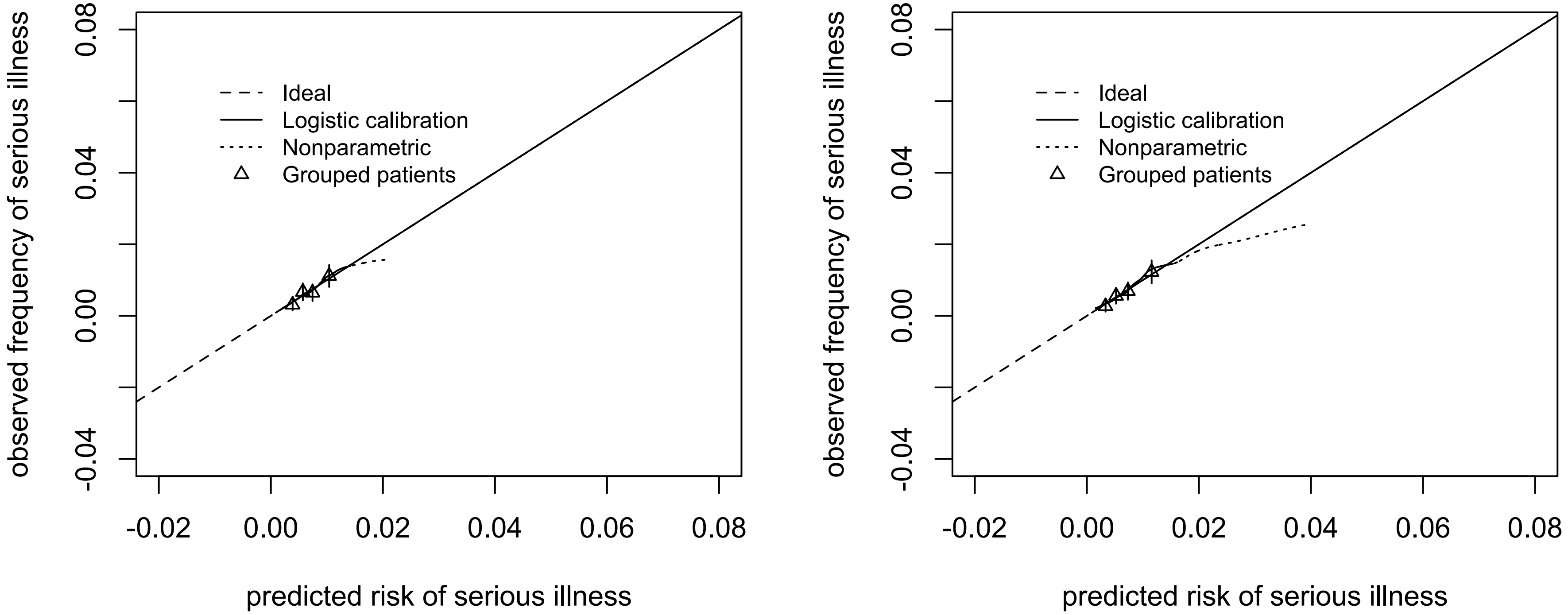

Supplement: S4 Fig — (PDF) [file pone.0254366.s011.pdf]
