# Supplementary material for: A clinical prediction model to identify children at risk for revisits with serious illness to the emergency department: A prospective multicentre observational study
Source: PLoS One. 2021 Jul 15;16(7):e0254366. doi: 10.1371/journal.pone.0254366 (PMC8281990; doi:10.1371/journal.pone.0254366)
Supplement: S5 Fig — (PDF) [file pone.0254366.s012.pdf]

S5 Fig. Bias corrected calibration plots

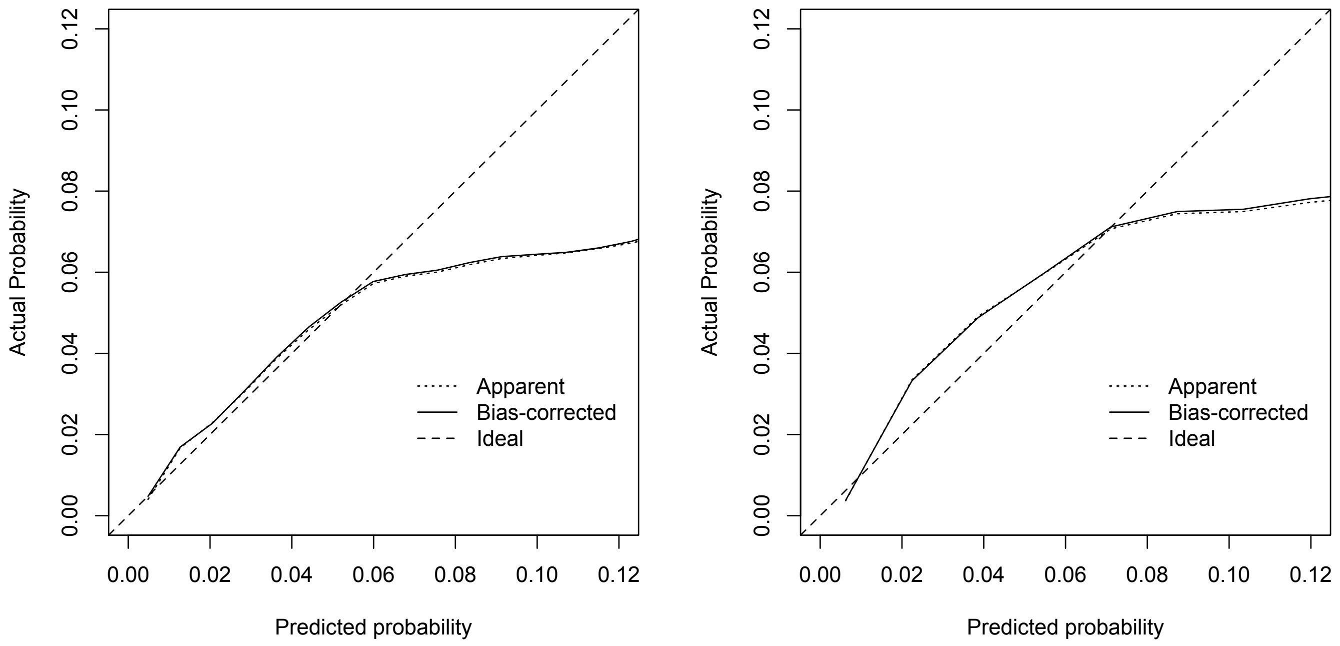

Legend:

These bias corrected calibration plots show the predicted risks (x-axis) and the observed frequencies (y-axis) of return visits with serious illness for both the clinical (left) and extended model (right) for the final clinical prediction models. The dashed diagonal line represents ideal calibration. The black lines are the bias-corrected calibration (bootstrap: n=200; {rms} library, {calibrate} function in R4.0.0), which mostly overlie the apparent calibration curves confirming goodness-of-fit. Mean corrected factor (clinical model): 0.003; mean corrected factor (extended model): 0.002. The suboptimal calibration for the higher predicted probabilities is largely due to a sparsity of cases, with good calibration for the range of predicted probabilities for most cases.
