# Supplementary material for: A clinical prediction model to identify children at risk for revisits with serious illness to the emergency department: A prospective multicentre observational study
Source: PLoS One. 2021 Jul 15;16(7):e0254366. doi: 10.1371/journal.pone.0254366 (PMC8281990; doi:10.1371/journal.pone.0254366)
Supplement: Attachment — Submitted filename: rebuttal v2 TRIAGE revisits PLOS.docx [file pone.0254366.s013.docx]

Dear editor,

Thank you for reviewing our manuscript, and for giving us the opportunity to reply to your and the reviewers’ comments. We think that the current version of the manuscript has improved thanks to the many valuable comments, and we hope to have sufficiently addressed and explained the issues raised by yourself and the reviewers.

We have performed substantial re-analyses following the reviewers’ comments. In particular, in reply to reviewers 2 and 3, we reconsidered our approach on how to best account for inherent differences between the hospitals in our study. Although this did not greatly impact the overall diagnostic performance and discrimination, the model showed better calibration, and hence we feel this model is better placed clinical applicability. As a result, we have updated the contents of Tables 3 – 5 , as well as Figures 2 – 3, and the Supplemental files 2 – 8.

Please find below our responses (in red) to each of the issues raised. Should there be any more outstanding questions, we will be more than happy to clarify these and to continue the discussion.

With kind regards,

Ruud Nijman

Corresponding author

Requests from the editors:

*Please structure your abstract using the PLOS Medicine headings (Background, Methods and Findings, Conclusions).

We have changed the structure of the abstract to include the PLOS Medicine headings.

*Ideally, in the last sentence of the Abstract Methods and Findings section, please include a brief note about any key limitation(s) of the study's methodology.

We included the following sentence in the abstract:

*As study limitations, variables on ethnicity and social deprivation could not be included, and only return visits to the original hospital and not to those of surrounding hospitals were recorded.*

*At this stage, we ask that you include a short, non-technical Author Summary of your research to make findings accessible to a wide audience that includes both scientists and non-scientists. The Author Summary should immediately follow the Abstract in your revised manuscript. This text is subject to editorial change and should be distinct from the scientific abstract. Please see our author guidelines for more information: <https://journals.plos.org/plosmedicine/s/revising-your-manuscript#loc-author-summary>

We have included this non-technical Author summary on p4 of the manuscript.

*Please remove the section after the abstract headed "What is already known" (as this is presumably for a different journal's format).

We have removed these headings from the revised manuscript.

*We'd suggest using the TRIPOD guideline (developed to support reporting of multivariable prediction models - <https://www.equator-network.org/reporting-guidelines/tripod-statement/>), or other appropriate reporting guideline, to support reporting in this study - if doing so we'd suggest noting the TRIPOD guideline was used in the Methods section, and then including a completed checklist as a supporting information file with the revised paper.

We have included a statement in the Methods section, and uploaded a completed TRIPOD checklist as a supplemental file.

*We'd also suggest clarifying in the Methods whether the analytical approach reported here was laid out in a prospective protocol or analysis plan? Please state this (either way) early in the Methods section.

a) If a prospective analysis plan (from your funding proposal, IRB or other ethics committee submission, study protocol, or other planning document written before analyzing the data) was used in designing the study, please include the relevant prospectively written document with your revised manuscript as a Supporting Information file to be published alongside your study, and cite it in the Methods section. A legend for this file should be included at the end of your manuscript.

b) If no such document exists, please make sure that the Methods section transparently describes when analyses were planned, and when/why any data-driven changes to analyses took place.

c) In either case, changes in the analysis-- including those made in response to peer review comments-- should be identified as such in the Methods section of the paper, with rationale.

To clarify, all data were collected as part of a prospective study with the main aim of improving triage systems for children. We have uploaded the original protocol and funding application of the TRiAGE study: a study primarily designed to validate and improve triage systems for children. The study sites tailored their data capturing systems in the emergency department specifically for the purposes for the TRiAGE study. Although the research question answered in this manuscript is very closely related to the original study objectives, with similar requirements for data collection and study design, we feel that framing our analysis as a ‘secondary analyses’ of the prospectively collected data is more accurate. We have changed this in the abstract and Methods section. We used additional up to date insights in clinical prediction modelling to optimise the original analytical plan. If the editor believes this is appropriate, we would gladly support making the protocol and (unsuccessful) funding application for this study publicly available.

Methods section, paragraph ‘Design, participants, and setting’, P6:

*We performed a secondary analysis of data collected as part of a prospective observational multicentre study in five European EDs, with data collected between 2012 and 2015*.

In addition, a manuscript using part of the data of the TRiAGE study with the aim to develop and validate an early warning score in the emergency department has now been accepted by the Lancet Adolescent and Child Health (DOI: <https://doi.org/10.1016/S2352-4642(20)30139-5>). This study addressed a different research question, but validates our used methodology. We have referenced to this publication in the Methods section.

We hope our answers to the questions the above provide all the necessary additional background information regarding study design, ethics, and data analysis.

-----------------------------------------------------------

Comments from the reviewers:

Reviewer #1: Thank you for the opportunity to review this interesting study on "A clinical prediction model to identify children at risk for revisits with serious illness to the emergency department: a prospective multicentre observational study".

This is a multi-center prospective observation study involving 98,561 pediatric patients who discharged from 5 different hospital EDs with subsequent serious illness occurred in 1,026 patients within 7 days from the index discharge. This study derived an interesting model (using four out of five ED data) to predict patient ED returns with serious illness and validated their model (using one of the five ED data). More importantly, authors identified model could be used as a "rule-in" criteria if patients were found with a calculated risk of >5% (LR+ of 5.88, specificity=98%). Meanwhile, same model can be used as a "rule-out" criteria if patients were found with a calculated risk of <0.5% (LR- of 0.25 and sensitivity of 92%).

Study enrolled a significant number of subjects, using reasonable statistical analysis including data imputation, internal validations, and is well written. However, this predictive model derived from the manuscript lacks clinical values and I personally believe it is hard to apply to the current clinical practice. My main concerns are as the followings

1. A relatively rare prevalence of return patients with serious illness, in this study, only 1.0% of patients considered as serious illness with ED returns. Given such low prevalence, your NPV will be high regardless. Using LR+ of 5.88, with pre-test probability of 1%, after using such criteria, will result in approximately 6% of post-test probability, which seems to have less clinical values. Similarly, using LR- of 0.25 with pretest probability of approximately 1%, this will result in an approximately 0.25% of posttest probability, in another word, using such criteria to "rule-out" serious illness ED returns, will decrease the initial 1% to 0.25% (4 fold decrease). With a rare prevalence, such decrease does not appear to be effective.

Additionally, in table 5, authors listed their sen/spe, PPV/NPV, LR+/LR-. If use risk>5% as the criteria to "rule-in" patients:

Serious Illness	Non-serious illness

Prediction tool (+)	92 (TP)	1,448 (FP)	1,540

Prediction tool (-)	934 (FN)	94,795 (TN)	95,729

1,026	96,243	97,269

With this prediction tool, we will have over 90% of FP patients. Though high NPV and LR+ will be yielded, pragmatically, using such criteria will result in over 90% of unnecessary admissions. Similarly, if use risk<0.5% as the criteria to "rule-out" patients, the sensitivity of 92% indicated that approximately 8% of patients will be missed. I don't think that ED physicians will feel comfortable to miss these number of patients. In addition, when adding patients who might be eligible to use such criteria, only 33% patients with calculated risks of <0.5% and only 2% of patients with calculated risks of >5%, in another word, the majority of the study patients cannot be used with this criteria.

We thank the reviewer for this carefully considered argument and for addressing the clinical applicability of the models. Importantly, we are not arguing that all children with a high risk of serious illness at a return visit should be admitted. This would be unfeasible and unwanted. Rather, we argue that this group of patients warrant careful safety netting or personalised follow-up, which we have hopefully clarified by adding more details to the Discussion section, paragraph on Implications for clinical practice and future research. How this follow-up should look is highly setting dependent, with some institutions having the capacity to organise planned face to face review appointments in ED or by an ambulatory care team, and in other settings this might be done via telephonic consultation. Many factors contribute to the development of serious illness, including natural progression and treatment compliance. By implementing tailored safety netting and follow-up this will help early detection of children with progressing disease, preventing delayed presentations after an initial health care visit, as well as ensuring treatments are continued at home, and are given in a timely and appropriate manner. Examples of the latter could include antibiotic tolerance in young children, appropriate inhaler technique of bronchodilators, and reinforcement of hydration strategies.

We appreciate the reviewer’s view on the clinical applicability of the models, and we agree that the high-risk threshold of 5% will only apply to a minority of children, including only a small proportion of true positive children. Hence, we have included the 3% and 4% thresholds in Table 5; and focussed on the 4% threshold in the Results section and the abstract. We believe that the 3% and 4% threshold values would identify a larger, clinically important proportion of the true positive children, whilst filtering out large numbers of true negative children, as exemplified by very reasonable diagnostic performance (see table below, extracted from updated table 5).

| Clinical  model | Risk threshold | N (%) | Sensitivity | Specificity | PPV | NPV | LR + | LR - |
| --- | --- | --- | --- | --- | --- | --- | --- | --- |
|  | >3% | 15,534 (16%) | 0.41 (0.38 – 0.44) | 0.84 (0.84 – 0.85) | 0.03 (0.02 – 0.03) | 0.99 (0.99 – 0.99) | 2.61 (2.42 – 2.82) | 0.70 (0.66 – 0.74) |
|  | >4% | 7,391 (8%) | 0.28 (0.25 – 0.30) | 0.93 (0.92 – 0.93) | 0.04 (0.03 – 0.04) | 0.99 (0.99 – 0.99) | 3.73 (3.37 – 4.13) | 0.78 (0.75 – 0.81) |
| Extended model | >3% | 13,222 (14%) | 0.42 (0.39 – 0.45) | 0.87 (0.86 – 0.87) | 0.03 (0.03 – 0.04) | 0.99 (0.99 – 0.99) | 3.18 (2.95 – 3.42) | 0.67 (0.63 – 0.70) |
|  | >4% | 7,060 (7%) | 0.28 (0.25 – 0.31) | 0.93 (0.93 – 0.93) | 0.04 (0.04 – 0.05) | 0.99 (0.99 – 0.99) | 4.00 (3.62 – 4.42) | 0.77 (0.74 – 0.80) |

The range of predicted risks (Figure 2, Table 5, S6) also indicate that clinical deterioration after a first ED presentation is difficult to predict. This reflects a well-known phenomenon in paediatrics, especially when children present early in the disease course (for example: Thompson et al., Lancet, 2006)(1). However, we should also consider that the absolute change from pre- to post-test possibilities will nearly always be low in the case of a relatively rare outcome.

In addition, the proposed intervention of tailored safety netting and follow-up can potentially be easy to implement and at low-cost, adding weight to the discussion of number needed to be screened. We don’t suggest that all revisits with a serious illness can be prevented by implementation of our model; and future studies designed for this specific purpose should look at the ability and net benefit of the model with safety netting and follow-up strategy, in combination with cost-effectiveness analysis.

In routine practice, many clinicians will already give safety netting advice, and more senior clinicians will likely have an experienced feel for those patients at highest risk for deterioration. Our models enable this for more junior physicians, who see the large majority of unwell children in acute care facilities, and for settings with no dedicated paediatric emergency care team, which is a very common scenario in most of Europe.

We added to the Results section, paragraph Clinical prediction model, p.11:

*7,391 children (8%) had a risk of >= 4%, which was useful for ruling in a revisit with serious illness, with positive likelihood ratio 3.73 (95% CI 3.37 – 4.13) and specificity 0.92 (95% CI 0.92 – 0.93) (table 5). Increasing this high-risk threshold to 5% or above resulted in improved ‘rule in’ value (positive likelihood ratio 4.81 (95% CI 4.15 – 5.57) and specificity 0.97 (95% CI 0.97 – 0.97)), but at the cost of being applicable to fewer children (n=3,270, 3%).*

We added to the Discission section, paragraph Implications for clinical practice, p14:

*How this follow-up should look will be highly setting dependent, with some institutions having the capacity to organise planned face to face review appointments in ED or by an ambulatory care team, and in other settings this might be done via telephonic consultation or with the involvement of primary care. Many factors contribute to the development of serious illness, including natural disease progression and treatment compliance. By implementing tailored safety netting and follow-up this will help early detection of children with progressing disease, preventing delayed presentations after an initial health care visit, as well as ensuring treatments are continued at home, and are given in a timely and appropriate manner. Examples of the latter could include antibiotic tolerance in young children, appropriate inhaler technique of bronchodilators, and reinforcement of hydration strategies.*

We added to the Discission section, paragraph Implications for clinical practice, p14:

*More senior clinicians will likely have an experienced feel for those patients at highest risk for deterioration. Our models enable this for more junior physicians, who see the large majority of unwell children in acute care facilities, and for settings with no dedicated paediatric emergency care team.*

2.	Authors mentioned briefly in their limitation, this study does not report how many patients who did return to the same EDs instead of seeking healthcare in other EDs, therefore, their ED returns with serious illness could be biased. We also don't know if patients who had returns with serious illness are willing to visit the same ED or they will seek second opinions from other EDs. Furthermore, lots of confounders could affect patient ED returns, authors only focused on demographics, initial presentation, vital signs, which might not be accurate to derive this prediction model. Patients/family with high psycho-social risks could be a major contributor (e.g. do patients having sufficient primary care follow-up, if 7-day was used for ED returns, we don't know whether follow-up care can be scheduled within 7 days by pediatricians, we also don't know patient insurance coverage, if no insurance coverage, do they have a high chance to use ED as the primary medical home? Does
patient/their family lack of transportation, so they have to use ambulance for transportations? etc.). We understand the challenge of including all potential risks into the model. However, with prospective study, important risks are feasible to collect and are required to be included.

We thank the reviewer for raising these issues. We will attempt to unpick them, and to address them one at a time.

Firstly, there are arguments regarding ‘confounding factors’, and how these could have influenced, or biased, our outcome measure, and we have addressed this below in point 1. We agree that confounding factors are highly relevant when determining the associations between predictor variables and an outcome. When discussing the performance of a clinical prediction model confounding factors are typically less of an issue.(2) The non-inclusion of confounding variables doesn’t demerit the validity of our findings or the conclusions with regards to our clinical prediction models, as argued in reply to point 2, although additional variables might have increased our predictive ability. Our reply to point 3 will hopefully help in better positioning the clinical prediction models in the European health care systems. Point 4 details why we didn’t include mode of transportation as another variable in the model.

1) ‘We also don't know if patients who had returns with serious illness are willing to visit the same ED or they will seek second opinions from other Eds’

We addressed this issue in more detail in reply to the comments of reviewer 2. Our study sites have a leading local and regional function role in providing urgent and emergency care to children. Although we can not rule out that some children re-presented to other EDs, this appears less likely for children with serious illness. For children within an acute disease episode, parents and carers are actively encouraged to return to the same ED in case of clinical deterioration; similarly, primary care physicians and emergency services will refer patients back to their local and treating ED rather than sending elsewhere, also as medical records are not readily shared between health care providers. In addition, we lack data to show the impact of revisits to other EDs than the one originally visited, and future studies will need to provide detailed insight into where children re-present during an episode of acute illness. At present, no clear data exist showing where and when children with an acute illness re-present in European countries. Future studies should look more into these type of network analyses, but this was beyond the scope of our study.

We added to the Discussion section, paragraph Limitations, p.16:

*In addition, we lack data to show the impact of revisits to other EDs than the one originally visited, and future studies will need to provide detailed insight into where children with a serious illness re-present during an episode of acute illness*.

2) ‘Furthermore, lots of confounders could affect patient ED returns, authors only focused on demographics, initial presentation, vital signs, which might not be accurate to derive this prediction model’

We agree with the reviewer that we included a relatively small number of predictor variables in our model. As discussed in the Methods and Discussion sections, we did so as these variables would be routinely collected for each patient in most EDs across health systems. Additionally, they have limited intra-user and inter-user variability, and can be collected homogeneously and in a systematic manner across sites. Hence, the included variables are least subjective and with minimal geographical variation. All this contributed to a model applicable to multiple settings, as is supported by the robust performance of the cross-validation studies. Social economic status, ethnicity and deprivation status were not included and these would potentially be informative, yet we were not able to obtain these because of ethical constraints in most European countries, as stated in the Limitations paragraph in the Methods section. Additional variables and their incremental predictive value should be studied in future studies, weighing the potential incremental predictive value against the arguments mentioned above.

We added to the Discussion section, paragraph Limitations, p16;

*Also, only a relatively small number of variables were included resulting in a low explained variance of our models, meaning future studies should explore the incremental predictive value of additional variables, such as social economic status and ethnicity. We did not consider language fluency, as reliably determining parental or carer’s language proficiency was not feasible.*

3) ‘do patients having sufficient primary care follow-up, if 7-day was used for ED returns, we don't know whether follow-up care can be scheduled within 7 days by pediatricians’

And

‘we also don't know patient insurance coverage, if no insurance coverage, do they have a high chance to use ED as the primary medical home’

We agree with the reviewer that several important factors such as primary care system and patient insurance coverage influence patient access to EDs and these factors are highly variable between countries. Importantly, in our European institutions, in the large majority of cases, hospital admission is warranted to provide therapeutic of supportive treatment that would not have been able in the community or by other health care providers. So, our chosen outcome measure is not directly influenced by the interplay of primary care, insurance status and ED accessibility and showed to be relatively robust across settings. In our updated analyses we also accounted for the differences between hospitals in an improved way, as highlighted by the improved calibration in the cross-validation studies.

We would like to address a number of important determinants of ED access mentioned by the reviewer. Across the sites included in our study insurance coverage, access to urgent and emergency care, and availability of primary care were similar. Hence, we did not include insurance coverage or other markers of national health systems in our model. To summarise the health systems:

a. UK: a publicly funded National Health Service with open access, free of charge, to primary care, and urgent and emergency care.
b. The Netherlands has universal health care, with all adults are required to have a basic health insurance; children are insured via their parents. Primary care is freely accessible; there is an own risk for most other healthcare (385 euros per year per adult in 2020).
c. Portugal has universal health care coverage via the publicly financed national health service.
d. Austria has a two-tier health care system in which all individuals receive publicly funded care and additional private care is available.

All countries in our study have primary care with general practitioners as well as primary care paediatricians (Austria, Portugal) available, free of charge. From our referral data from four sites (see table below), it is clear that even in the presence of functioning primary care systems, there are great differences in how parents and their children access health care for urgent and emergency care; e.g. in the Lisbon cohort no patients were referred by general practitioners, whereas in the Netherlands close to 50% of cases were referred. Importantly, our models performed well across the sites.

|  | Erasmus cohort | Maasstad cohort | London cohort | Lisbon cohort |
| --- | --- | --- | --- | --- |
| Self | 5920 | 2644 | 11043 | 44272 |
| GP | 2365 | 4021 | 186 | 0 |
| Emergency services | 793 | 335 | 345 | 1526 |
| Other | 4060 | 650 | 1369 | 0 |

We suggest finding local solutions for the best solutions to follow-up and monitoring of children after their initial ED discharge. For example, in the London setting the ED team have an ambulatory outreach team available for additional reviews by telephone or in person. In other settings, close collaboration with the GP might be a better alternative for organising tailored follow-up for high risk patients. As outlined earlier, we added to the Methods section, paragraph Implications for clinical practice and future research, p.14.

We added to the methods section, paragraph Limitation, p.16:

*As our study hospitals have an important regional referral function with limited admission capacity, hospital admission was warranted only to provide therapeutic of supportive treatment that would not have been able in the community or by other health care providers in the large majority of cases. Although some children might have had a lower threshold for admission at revisit, as well as potential social reasons, this will only represent a small proportion of the children admitted at revisit.*

4) ‘Does patient/their family lack of transportation, so they have to use ambulance for transportations’

We did not include this in our analysis, because these data were not available for the Vienna cohort. Also, for those sites with the data available, mode of arrival, i.e. the use of ambulance and emergency services, was not associated with outcome of patients at initial visit. We would also like to argue, based on for example our experience in the MOFICHE study (3), that in Europe the use of emergency services was not just related with severity of disease but also with health systems and the local culture of accessing health care. In an additional analysis (i.e. unadjusted logistic regression model for referral status and our outcome of interest), the use of ambulances for initial transportation doesn’t increase risk of revisit compared with GP referrals.

|  | B | S.E. | Wald | df | Sig. | Exp(B) |
| --- | --- | --- | --- | --- | --- | --- |
| Source of referral: reference group is Other |  |  | 347.213 | 3 | .000 |  |
| Source of referral(1): Self | -1.345 | .088 | 232.866 | 1 | .000 | .261 |
| Source of referral(2): GP | -.086 | .107 | .642 | 1 | .423 | .918 |
| Source of referral(3): emergency services | -.887 | .182 | 23.775 | 1 | .000 | .412 |
| Constant | -3.495 | .076 | 2122.498 | 1 | .000 | .030 |

Overall, this is an interesting study with large sample size. However, to draw your conclusion, and make your model applicable to the clinical practice require more robust analyses. Currently, I cannot see such accuracies, I will recommend this study might require significant revisions before suitable for publication.

Again, we thank the reviewer for raising the important issue of applicability. In short, we completely agree with the reviewer that multiple patient, setting, and health care system related factors could influence the risk for a revisit with a serious illness. We have chosen a subset of these predictors, that are routinely collected, objective determinants, without the need for additional ethical approval. Through our cross-validation by study site approach, we have shown that our model remains robust across settings. Based on the reviewer’s comments we have further emphasized in the discussion section the existence of multiple additional confounders. Further studies are welcomed that address these and assess whether they can improve predictive performance of our model.

Reviewer #2: Abstract

Please put the time frame for recruitment in your abstract and paper.

We have included this in the abstract.

Methods

Please justify the inclusion of only <16 yo children.  At least in the United States, 18 and 21 are more common cut-offs (Dobson, 2002).

All our study centres only saw children up to 16 years of age in dedicated paediatric emergency departments. The upper age limit varies across Europe, with most centres seeing children up to 16 years, with a minority seeing children up to 14 years or 18 years (Hagedoorn et al., PLOS Medicine, 2020)(3).

Please justify the use of 7 days for return visit (many studies have used 24, 72 hours; for adults, one study suggested up to 9 days (Rising, 2014).

We thank the reviewer for this remark, and we like to use this opportunity to highlight the shift in focus from many studies looking at any (and mostly avoidable) return visits to a focus on those with a serious illness. Less evidence exists for the optimal follow-up time window for this outcome in children. For some groups of patients, such as children with respiratory, febrile, and gastrointestinal illnesses, studies have suggested time windows of up to 5 days (de Vos - Kerkhof et al., ADC, 2016; de Vos – Kerkhof et al., Eur J Pediatr, 2018)(4,5); to allow for a more diverse group of diagnoses we opted for 7 days to allow for sufficient time of progression of disease symptoms.

Can you separate out hospital admission from PICU/death?  In particular, it seems often times we have a lower threshold to admit on the second visit so some children may get admitted *because* they have a return visit, which would not necessarily be a marker of worsening disease.  Understanding if the patterns for PICU/death are different would also help understand the risk in this group.

We couldn’t construct a separate model for critical care admission or death at revisit, as the numbers for this were too few. Despite having significant overall patient numbers, we only had 23 children admitted to PICU at revisit. As described in S2 Appendix 2, the clinical signs and symptoms of these children varied widely. Children with shortness of breath and children with fever were relatively overrepresented, but this still accounts for a small number of children.

Secondly, our hospitals have an important regional referral function with limited admission capacity. Hospital admission is warranted only to provide therapeutic of supportive treatment that would not have been able in the community or by other health care providers in the large majority of cases. Although some children might have had a lower threshold for admission at revisit, as well as potential social reasons, this will only represent a very small proportion of the children admitted at revisit. Importantly, with only a very small number of children who died or who were admitted to PICU, there is no other valid golden, unifying standard for serious illness. As shown in our secondary analysis with a modified outcome (Discussion section, paragraph Limitations, p.16), also including children with lifesaving interventions and highest triage urgency, the total number of cases only added 16 cases. Hence, to ensure applicability and validity of the model in a wide range of centres, we opted for an inclusive, uniform outcome measure. We felt duration of admission would not add to our outcome measure for all serious illness, as many hospitalised children receive necessary treatment with excellent clinical improvement within 24 hours (e.g. rehydration in children with vomiting and diarrhea, and treatment with steroids and bronchodilators in children with wheeze/asthma).

We added to the methods section, paragraph Limitation, p.16:

*As our study hospitals have an important regional referral function with limited admission capacity, hospital admission is warranted only to provide therapeutic of supportive treatment that would not have been able in the community or by other health care providers in the large majority of cases. Although some children might have had a lower threshold for admission at revisit, as well as potential social reasons, this will only represent a very small proportion of the children admitted at revisit.*

I am concerned about the lack of inclusion of the hospital as a predictor variable. Please explain how your method accounts for potential clustering by site.  This is critically important as it has the potential to meaningfully impact your results.

We thank the reviewer for raising this important point. We had originally included the hospital as a weighting variable in the logistic regression analyses, and not as a separate predictor variable. We agree that this method did not deal with the clustering per site enough. We have now performed re-analyses including the hospital as a categorical variable. For the cross validation studies, this means that we performed regression analyses including all model variables and the hospital variable (for 4 sites), and then adjusted the intercept using the linear predictor without the hospital variable for the validation studies using the fifth validation cohort. Similarly, for the final prediction models, we ran the models for all five cohorts including all model variables and a hospital variable, and then modified the intercept to represent the ‘average’ hospital. We also used this methodology in another recent paper (Zachariasse et al. Lancet Child & Adolesc, 2020)(6) Although this did not affect the discrimination of the model much, it impacted on the individual predictor variables, it slightly increased the average risk predictions, and considerably improved calibration. As a result, we have updated the contents of Tables 3 – 5, as well as Figures 2 – 3, and the Supplemental files 2 – 8.

Please justify the "leave-one-out" approach rather than combining the cohorts and using a technique such as 10-fold cross validation.

We thank the reviewer for the opportunity of elaborating on our choice of the methodology for the validation studies. Firstly, external validation aims to assess a model’s performance in different settings than where the model was derived.(7) We used internal-external validation, or ‘leave-one.-out’ approach, which is a validated, modern, and efficient approach.(8) This means, we derived the model in four settings and validated the model in a fifth setting that was left out of the model development, and we repeated this procedure five times. This method is considered to be an efficient validation strategy because it uses all available information, avoids wasting data by random splitting data in train and test sets, and illustrates heterogeneity in performance.(7–11) We have provided some references here to strengthen our argument for using this approach (we also included one in the original text).

Please clarify if triage status is coded the same at all of your hospitals? Why were co-morbidities only coded in 3 of the hospitals?

We can confirm that all hospitals used the Manchester Triage System. This is the most commonly used triage system in Europe. In reply to one of the comments from the editor, we have also provided a study protocol describing this in detail.

We were only able to code co-morbidity of three out of five hospitals as we encountered technical difficulties with the electronic data extraction for the comorbidity variable for these two hospitals. Because each country has its own healthcare system, hospitals differ as to how and whether diagnoses are systematically coded. For example, most European hospitals do not routinely use ICD-codes for billing purposes. The two hospitals without co-morbidity only had information on underlying chronic conditions available in free text fields. We did not consider it feasible, also because of the low prevalence of complex comorbidity in these hospitals, to manually recode these data fields.

Where are the characteristics of the children admitted to the PICU described? (you say "separately" but it is not clear if this is another manuscript and if so should it be cited here).

We have described the characteristics of the children admitted to PICU in S2 Appendix 2. In the Results section, paragraph Rates of revisits, p9, this is explained as follows:

*Twenty-three children (0.02%) were admitted to PICU and one child died at revisit (characteristics of PICU patients presented in S2 appendix 2).*

We have also added the reference to S2 Appendix 2 in the Methods section, paragraph Outcome measures, p8.

Results

Given that the CI for tachycardia overlaps 1, it is not significantly associated with a revisit; please rephrase. Similarly, many of the other reported CI include 1, which would be a non-significant OR.

We thank the reviewer for pointing this out. In our original manuscript, there was a difference between the Odds Ratios in Table 2 (including hospital variable) and Table 4 (without hospital variable). In this revised version, we included the hospital variable in the modelling of our prediction models, meaning T2 and T4 align better. There are still minor differences between the ORs in T2 (showing associations) and T4 (showing prediction model), as a result of how the hospital variable was used in the prediction model modelling, as detailed above. If the ORs of Table 2 are confusing, we would be suggest to remove these, and the p values, from Table 2 altogether.

Discussion

It seems you were unable to account for return visits to different centers; can you comment in limitations on how that might have impacted your results (as those return visits will be invisible to your study)?

We thank the reviewer for this valid point, and we have also addressed this issue in reply to reviewer 1. It is important to highlight that our study looked at serious illness only and not just any revisit. There will still be a risk that parents and carers will have sought care at another hospital than the one they originally visited. However, all our study hospitals serve a regional function providing specialist paediatric urgent and emergency care, and are widely regarded as leading centres in their region. Although we can not ascertain that patients will have been admitted to other hospitals, we deem this risk small and only applicable to an absolute minority of patients, as also argued earlier and as added to the Methods section, paragraph Limitations, p.16.

Is it possible that the differential rates of return visits to hospitals in your study are driven by the number of competing hospitals around them?  If not, I would like to know what you think is driving the variation between hospitals?

In our study, we saw higher rates of return visits with serious illness in the two Dutch cohorts, and lower rates in the Lisbon and Vienna cohorts. Generally, the hospitals included in the study have an important local function in delivering urgent and emergency care to children. In the included European health systems, there is no financial incentive for parents and carers to go to another hospital, and returning to the hospital that is responsible for current care of a child is widely encouraged. Hence, the differences are better explained by a number of factors other than competing hospitals:

1) The two Dutch cohorts (the Maasstad and Erasmus cohorts) had a significantly higher number of children with underlying comorbidities and patients requiring subspecialty care, which are risk factors of return visits with serious illness.
2) In the two Dutch cohorts, we had a higher percentage of children referred by the GP for their first ‘index’ visit. This implies that the GP filters out a large volume of non-urgent children at very low risk of revisit with serious illness, whereas these children continue to present to the EDs in the other 3 countries, leading to a lower incidence of revisits with serious illness.

Overall, we feel that the differences between the included cohorts can be explained by case mix differences as a result of the differing health systems and the population that they serve. The cross-validation studies reassuringly confirm that, by showing robust calibration performance, model performs well despite these health system differences. It is also worth noting that the range of reported outcomes is still at the lower end of what previously has been reported (in what are mostly US and South-East Asian studies).

It is important to recognize that some of the return visits in your outcome are actually a marker of good care (e.g. excellent return instructions for non-specific abdominal pain rather than imaging everyone to evaluate for appendicitis on the first visit).

We thank the reviewer for this comment, and we agree that these type of return visits, even when resulting in additional treatments and diagnostics, can be a marker of good clinical care. More and more, a watchful waiting approach is justified in acute paediatric conditions, on the pre-condition of high-quality safety netting. Hence, the example of appendicitis is precisely why our model can be useful: to recognise the potential for clinical deterioration once discharged from the hospital, allowing for detailed safety netting and tailored follow-up.

We added the following to the Discussion section, paragraph Implications for clinical practice and future research, p.15:

*Our prediction model can complement decision making in acute paediatric conditions where a watchful waiting approach is justified and a return visit can be interpreted as a marker of good clinical care, such as in children with non-specific abdominal pain deemed to be at low risk for appendicitis at first assessment.*

Can you comment in more detail on the exclusion of language from your predictive variables?  I understand that the inclusion of SES and health literacy may be complex and not well captured in existing administrative data, but language usually is. Multiple studies have shown that patients speaking minority languages (often Spanish in the US context) have higher rates of short-term return visits (see, for example, Samuels-Kalow, 2017).

We recognise that language proficiency plays an important role in delivering appropriate safety netting advice as well as understanding ways for accessing health care. Hence, language can play a role in both preventing unnecessary revisits, as well as ensuring timely recognition of clinical deterioration and continuation of appropriate treatment at home. Indeed, language was not part of our prospective data collection. We believed that assigning a first (or native) language does not necessarily reflect national/regional preferred language proficiency of the parent/carer; reliably determining the language proficiency was not feasible as part of our study design.

We added to the discussion section, paragraph Limitations, p.17:

*We did not consider language fluency, as reliably determining parental or carer’s* *language proficiency was not feasible.*

p.12 I would consider rephrasing line 16 "refusal…failure" as it sounds quite judgmental. Consider "inability" or "non-adherence to"

We have changed this according to the reviewer’s valuable suggestion.

I appreciate the focus on the implementation for safety netting advice and follow-up and not on "avoidable" revisits which are challenging to define consistently across sites.

We thank the reviewer for highlighting this important issue, and we agree that this differentiates our work from the already available literature.

Other notes—the answer to the data availability policy does not seem to fully address the PLOS requirements.

We are willing to change our data availability statement to ensure this is in line with PLOS Medicine requirements, and we will ensure access to the data used for this study. We have changed our data sharing statement accordingly and hope that this now fulfils PLOS Medicine standards.

-----------------------------------------------------------

Reviewer #3: "A clinical prediction model to identify children at risk for revisits with serious illness to the emergency department: a prospective multicentre observational study" aims to develop and validate a prediction model/calculator to identify children at risk of having a serious illness within 7 days of an index visit, as determined by a revisit to hospital. This objective is stated to be distinct from the more-usual objective in the literature of predicting revisits among child patients (whether due to serious illness or not). Logistic regression-based prediction models were leave-one-out cross-validated on data from five European emergency department (ED) sites, after which a final prediction model utilizing all available data was constructed. Prediction AUCs ranging from 0.62 to 0.75 were obtained for the cross-validation cohorts.

Methodologically, the study appears well-designed, with the relatively robust cross-validation results supporting the potential general utility of the prediction model (though predictive ability is moderate, from the reported AUCs). The final linear prediction models are fully described by their variables and coefficients in S7 Appendix 7, which should allow for replication. The set of variables examined also seems reasonable,  with some excluded variables (e.g. ethnicity, deprivation scores) generally unavailable in the European context, and others (e.g. socioeconomic status) argued to be possibly uninformative. The major remaining concerns then relate to the details of the statistical modelling, which do not appear to be comprehensively described:

We thank the reviewer for these supportive comments. Below, we tried to answer the raised issues as accurate as possible.

1. It is stated that the prediction models were developed using logistic regression based on a stepwise approach (Page 8). It is assumed that this stepwise approach selects variables from the original preselected set (Table 2), to the final reduced set of variables (Table 4; S7 Appendix 7). If so, the stepwise approach might be explained in greater detail.

We thank the reviewer for these remarks and we will use this opportunity to clarify these issues more.

Table 2 describes the characteristics of our combined cohorts, for cases and non-cases. Logistic regression analysis was performed to show association between these characteristics and our outcome measure of interest, using the model described in the legend of the table. In this single model all variables were included, including a hospital variable, any laboratory tests performed, any imaging performed, and any iv fluids and/or medications given; no stepwise regression was applied to this model. Some variables in Table 2 were not used for the multivariable modelling as a result of high percentage of missing values (e.g. blood pressure) or missing variable for a particular hospital (e.g. referrer), as also outlines in the legend of table 2.

The stepwise regression was only applied to the clinical prediction models. This means that we derived a clinical model in the first step, and then derived an extended model in the second step. During this process no variables were selected or excluded based on significance, and all variables were selected based on previous literature. This is reflected in Table 4, and in the text in section Results, paragraph Clinical prediction model, p.11. We added the following to the Methods section, paragraph Statistical analysis, p8:

*All variables were pre-selected and were included independent from their statistical contribution to the models based on previous literature, and not variables were excluded from the final prediction models based on statistical contribution.*

2. Likewise, it is stated that model calibration was performed through calibration coefficient/change in intercept via random effects models using the metafor package (Page 9). Again, these random effects models and the significance of the calibration coefficient/intercepts might be explained in greater detail, possibly extending the supplementary material for S5 Appendix 5/6 if appropriate.

Thank you for raising this point. We have provided additional information in the Methods section, paragraph Statistical analysis, p.9:

*External validity of the model was determined by estimating the discrimination by means of the Area Under the operating characteristic Curve (AUC), and model calibration by means of calibration plots, the calibration coefficient, or calibration slope, and change in intercept of the model. Calibration refers to the level of agreement between predicted risks and observed outcome; the ideal calibration coefficient, or slope, being one, indicating perfect agreement between observed and predicted risks. The calibration intercept reflects the extent that predictions are systematically too low or too high (‘calibration-in-the-large’), e.g. due to differences in the underlying incidence between cohorts, with an optimal value of zero.*(12) *The five estimates of AUCs and calibration coefficients for each of validation cohorts were then pooled by means of random effects models, using metafor package in R, version 3.6.1, to provide summary estimates. Finally, we developed a clinical prediction model combining all available data from the five cohorts and with a refitted intercept to allow for hospital differences, and an apparent overall AUC was calculated.*

We hope this clarifies the role of calibration in the validation of prediction models, which we agree with the reviewer, might not necessarily be intuitive and clear for your readership. We chose to add this to the main body of the text, but we would consider moving this to the S5 and S6.

3. The "weighted variable" referred in Page 8, Line 9 might be clarified. Does this relate to the calibration? This is because there appears no further mention of the weight(ed) variable, other than a reference in the Limitations section.

In our original analysis, we included the weighted variable to account for the different cohort sizes and account for between-cohort differences. We have now, as discussed in answer to a comment raised by reviewer 2, performed new analyses, using hospital as a categorical cohort to better account for differences between the cohorts. This has been modified throughout the Methods section, paragraph Statistical Analysis. As a result, we have updated the contents of Tables 3 – 5 , as well as Figures 2 – 3, and the Supplemental files 2 – 8.

4. For the multiple imputation model (Page 9), it might be explicitly clarified as to whether there being 25 databases, was due to the imputation depending on different available data (e.g. for the Erasmus cohort, the Erasmus data was imputed based on itself, while the remaining training data was imputed based on the other four hospitals, and so on)

We thank the reviewer for this comment. We imputed all data simultaneously, using a hospital variable to differentiate between the different hospitals. We have modified this in the Methods section, paragraph Statistical Analyses, p8:

*Missing data for vital signs (table 2) were imputed using a multiple imputation model including a hospital variable, age, available vital signs, triage urgency, presenting problem, and discharge destination. This resulted in twenty-five iterative databases, including data from all five cohorts, on which we performed statistical analysis after which results were pooled.*(13) *Imputation was performed using the MICE imputation package in R, version 2.15.2.*

5. The AUC for the combined cohort (Page 11) might also be mentioned/plotted for both the clinical and extended model, to facilitate comparison with the cross-validation cohorts (Table 3; calibration coefficient/intercept assumed not to be relevant for combined cohort). It is recognized that validation of a model developed with the combined cohort on itself might advantage the model to some extent, since it has access to all validation data.

We thank the reviewer for this comment. Although we had originally decided against including an apparent overall AUC for both models, we have now included this in the Results section, paragraph clinical prediction model, p11:

*The clinical model based on all cohorts combined had an apparent AUC of 0.73 (95% CI 0.71–0.74); the extended model also including intravenous medications and/or fluids, imaging, and laboratory investigations had improved AUC of 0.74 (95% CI 0.72–0.75; LR test for overall model performance improvement, p <0.001).*

Minor comments:

6. The sentence describing imputation for missing data beginning Page 9, Line 8, might be moved to the paragraph ending Page 8, Line 22, before the prediction modelling is done proper. This is because the imputation would have been done before the modelling, to the best of our understanding.

We thank the reviewer for this suggestion, and we have moved this paragraph accordingly.

7. For the various clinical variables, were they extracted from EHR based on specific codes? If so, these codes might be listed in the supplementary material to maximize replicability.

For the purposes of this study, we did not use specific EHR based codes. Importantly, each of the study sites incorporated the pre-defined data collection in their routine clinical practice in a way that best aligned with their local, existing data collection systems, and in the local language spoken.

All sites used the same triage system, i.e. the Manchester Triage Systems as outlined in the Methods section. We added a sentence to better clarify the coding of the presenting symptom, which was based on any of 52 selected flowcharts of the Manchester Triage System. And we added the coding to Appendix 1.

Section Methods, paragraph *Data collection and predictor variables*, p7:

*These flowcharts were re-classified into 11 main groups of presenting symptoms (S1 Appendix 1).*

8. For Table 5, certain figures might be given to more decimal places, to facilitate verification of derived values (e.g. For Clinical model >5%, LR+ computed from sensitivity=0.09 and specificity=0.98 is 4.5, instead of the given 5.88, probably due to rounding)

We can confirm that the derived values as shown in table 5 are correct (even though now updated with our renewed analyses), and that, as also suggested by the reviewer, the recalculated LR+ in the example given is due to rounding differences.

We fully agree with the reviewer on the importance of decimals, and we, for example, provided coefficients with 3 decimal values in S7 to allow for more accurate calculations. However, we would like to present diagnostic performance measures in table 5 in the conventional manner and use 2 decimals. However, if the editor feels it is appropriate to provide an additional decimal, and that this is line with PLOS Medicine house-style, we can readily provide the numbers with 3 decimals.

9. It might be briefly discussed whether the overall prevalence of serious illness (1.0% for the combined cohort, from Table 5 Legend) might affect the performance of the model in a new setting (since this is also listed as an assumption in the digital calculator, from Figure 3). This might be a pertinent limitation, separate from COVID-19 impact.

If the incidence of serious illness on return visit is known, then a model can be adjusted using calibration in the large. Currently, our digital calculator doesn’t account for this yet, but this will be part of ongoing research activities. Importantly, the model performed well across the validation studies despite different underlying incidence rates.

We added to the Methods section, paragraph Implications for clinical practice and future research, p.14:

*The differing underlying incidence of serious revisits, as also reflected in the intercept values of the cross-validation studies (‘calibration-in-the-large’,* *table 3), indicate that the local incidence should be taken into account when interpreting the risk predictions. If the local incidence is known, one could adjust our model for local use by adjusting the intercept of the model.*

10. Given that this is a predictive task, the authors might consider attempting machine learning techniques in future work.

We thank the reviewer for this suggestion, and we have added a statement on machine learning in the Methods section, paragraph Implications for clinical practice and future research, p14:

*In addition, the potential for machine learning techniques to improve our prediction model should be explored.*

1.	Thompson MJ, Ninis N, Perera R, Mayon-White R, Phillips C, Bailey L, et al. Clinical recognition of meningococcal disease in children and adolescents. Lancet. (2006);367:397–403.

2.	Steyerberg EW. Clinical Prediction Models: Application of Prediction Models [Internet]. Springer Science + Business Media. Springer-Verlag New York; (2009);101–111. Available from: http://www.springerlink.com/index/10.1007/978-0-387-77244-8

3.	Hagedoorn NN, Borensztajn DM, Nijman R, Balode A, von Both U, Carrol ED, et al. Variation in antibiotic prescription rates in febrile children presenting to emergency departments across Europe (MOFICHE): A multicentre observational study. PLOS Med [Internet]. (2020);17:e1003208. Available from: https://doi.org/10.1371/journal.pmed.1003208

4.	De Vos-Kerkhof E, Geurts DHF, Wiggers M, Moll HA, Oostenbrink R. Tools for “safety netting” in common paediatric illnesses: A systematic review in emergency care. Arch Dis Child. (2016);101:131–9.

5.	De Vos-Kerkhof E, Geurts DHF, Steyerberg EW, Lakhanpaul M, Moll HA, Oostenbrink R. Characteristics of revisits of children at risk for serious infections in pediatric emergency care. Eur J Pediatr. (2018);177:617–24.

6.	Zachariasse JM, Nieboer D, Maconochie IK, Smit FJ, Alves CF, Greber-Platzer S, et al. Development and validation of a Paediatric Early Warning Score for use in the emergency department: a multicentre study. Lancet Child Adolesc Heal [Internet]. (2020);4:583–91. Available from: https://doi.org/10.1016/S2352-4642(20)30139-5

7.	Riley RD, Ensor J, Snell KIE, Debray TPA, Altman DG, Moons KGM, et al. External validation of clinical prediction models using big datasets from e-health records or IPD meta-analysis: opportunities and challenges. BMJ [Internet]. (2016);353. Available from: https://www.bmj.com/content/353/bmj.i3140

8.	Steyerberg EW, Harrell FEJ. Prediction models need appropriate internal, internal-external, and external validation. J Clin Epidemiol. (2016);69:245–7.

9.	Steyerberg EW. Validation in prediction research: the waste by data splitting. J Clin Epidemiol. (2018);103:131–3.

10.	Royston P, Parmar MKB, Sylvester R. Construction and validation of a prognostic model across several studies, with an application in superficial bladder cancer. Stat Med. (2004);23:907–26.

11.	Siontis GCM, Tzoulaki I, Castaldi PJ, Ioannidis JPA. External validation of new risk prediction models is infrequent and reveals worse prognostic discrimination. J Clin Epidemiol. (2015);68:25–34.

12.	Steyerberg EW, Vickers AJ, Cook NR, Gerds T, Gonen M, Obuchowski N, et al. Assessing the performance of prediction models: A framework for traditional and novel measures. Epidemiology. (2010);21:128–38.

13.	Donders ART, van der Heijden GJMG, Stijnen T, Moons KGM. Review: A gentle introduction to imputation of missing values. J Clin Epidemiol. (2006);59:1087–91.

Additional reviewer’s comments following resubmission: dd. 20-10-2020

Reviewer #1: Authors addressed my concerns appropriately especially within the European healthcare system. Therefore, this manuscript is suitable for publication.

We thank the reviewer for the valuable comments helping us to improve the manuscript.

Reviewer #2: Thank you for this very careful response to review.  I have a few remaining questions.

1. P. 17 limitations section
   I do not think that you can say that this will only "represent a small proportion of the children admitted at revisit" unless you have data to back it up.  I would just say this is a limitation that should be addressed in future work (not for this paper, but you could do a sensitivity analysis testing different return visit assumptions and see how much it changes your results, especially around the question about children returning to hospitals that are not in your dataset).

We thank the reviewer for this suggestion, and we have changed the limitations section on p17 accordingly.

2. P. 17 limitations section
   Please remove "ethnicity" as one of the potential factors, as it is unlikely to be independently associated with your variable of interest once you assess SES and language.

We have changed this in the revised manuscript.

3. The adjustment for hospital is adequate although a mixed effects approach would be preferable.  If you are going to include the hospital variable in the model, I believe you may end up with incorrect standard errors if the cluster variable (hospital) is correlated with other covariates. At a minimum, one would probably need to include robust standard errors.

We thank the reviewer for these suggestions. Following discussion with our statistical team, we decided to keep our current methods of deriving and validating the prediction model, in line with our previous methodologically as applied in the paper by Zachariasse et al, published in the Lancet Child and Adolescent Health in August 2020. Of note, we did use mixed effects model to best estimate the AUC and calibration slope.

Reviewer #3: We thank the authors for addressing most of our concerns from the previous review round. However, it remains that more details might be provided on the "logistic regression based on a stepwise approach".

In particular, taking "logistic regression based on a stepwise approach" to mean "stepwise regression" (as stated in the authors' response), the initial set of all available variables is reduced to a smaller set of variables that is actually used in the final model (taken to be S7 Appendix 7); if so, the general practice would be to describe the initial set of all available variables, and also describe the method used to select important variables (e.g. forward selection, backward elimination, bidirectional elimination).

Moreover, the response appears to state that variables in Table 2 were dropped due to high percentage of missing values/missing variable for particular hospitals, and that stepwise regression was only applied to clinical prediction models (once the above variables had been dropped). However, it is further stated that "no variables were excluded from the final prediction models based on statistical contribution".

The authors might therefore clarify where the variable selection implied by "stepwise regression" takes place, assuming we have understood this properly; alternatively, if a stepwise regression has been used but no variables have been dropped, this might also be explicitly stated.

We appreciate these valid arguments from the reviewer. We see that our models do not necessarily fulfil the true definition of stepwise logistic regression models, as rightfully outlined by the reviewer. Rather, we used the term to explain we modelled two different models: one basic model using clinical variables available at triage, and one more extensive including diagnostics and treatment. W excluded variables with high number of missing before the modelling process. We have changed some of the wording in line with the reviewer’s last suggestion in the methods section.
